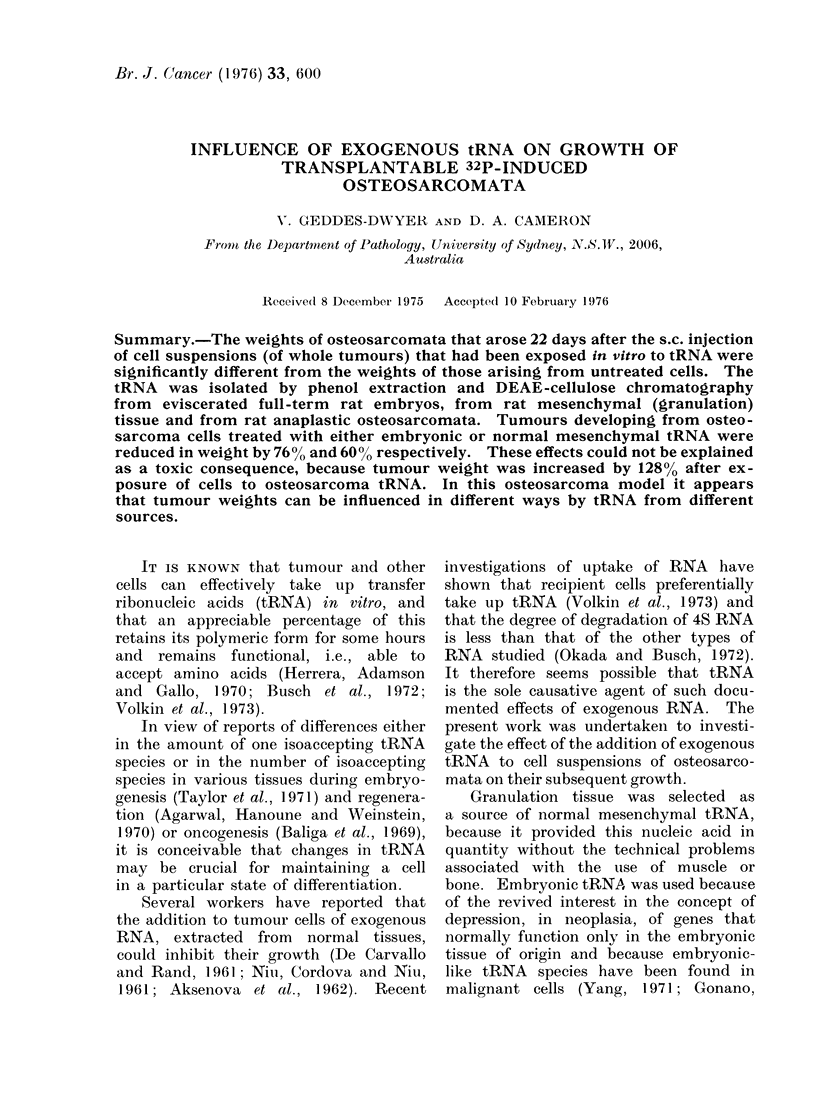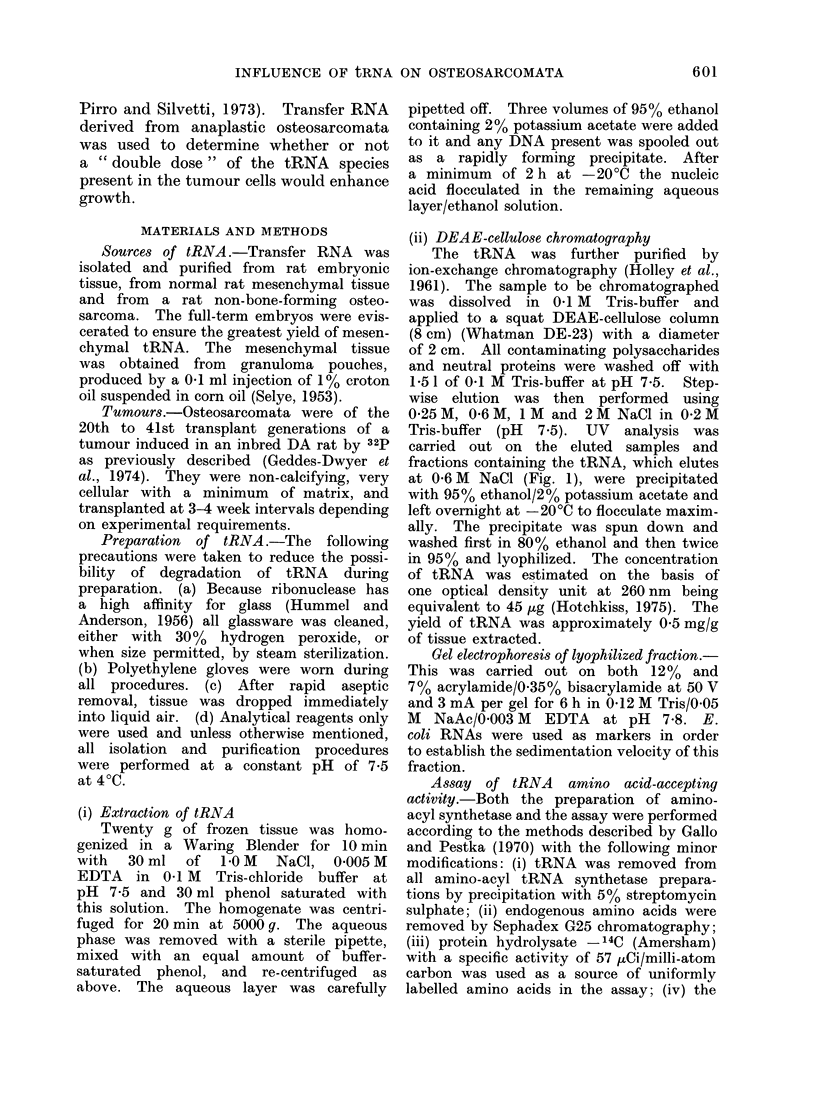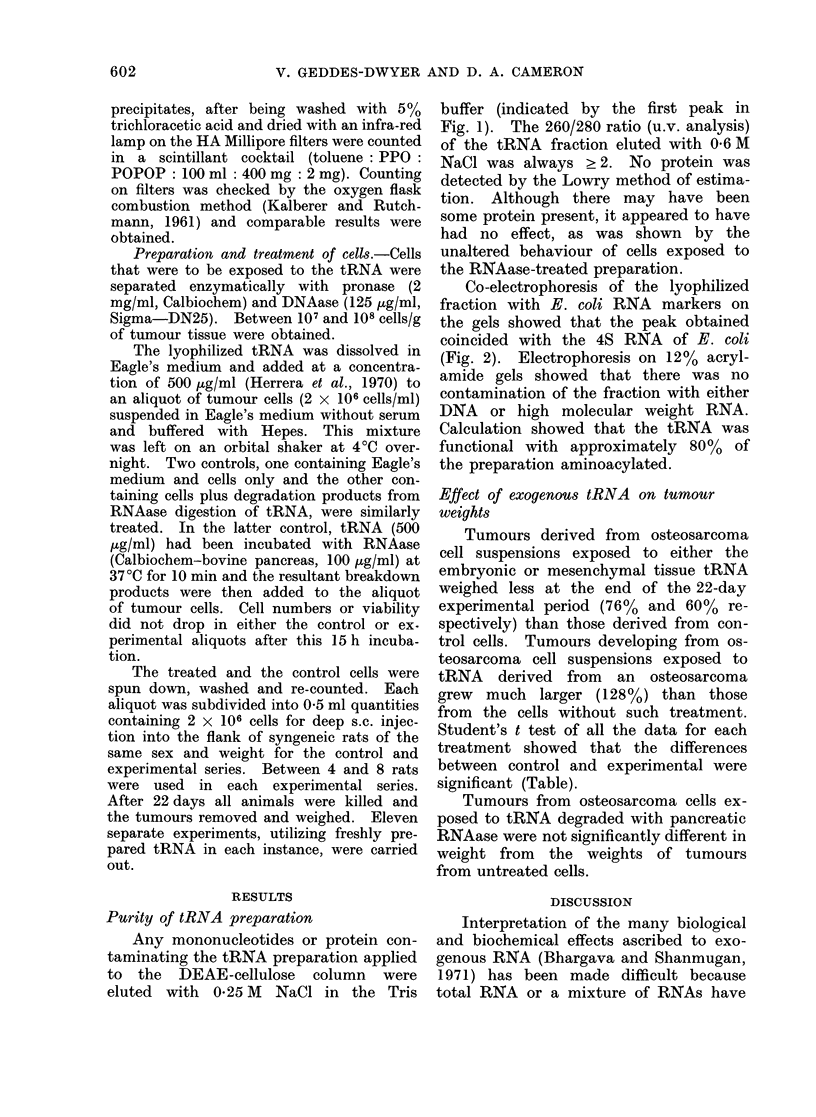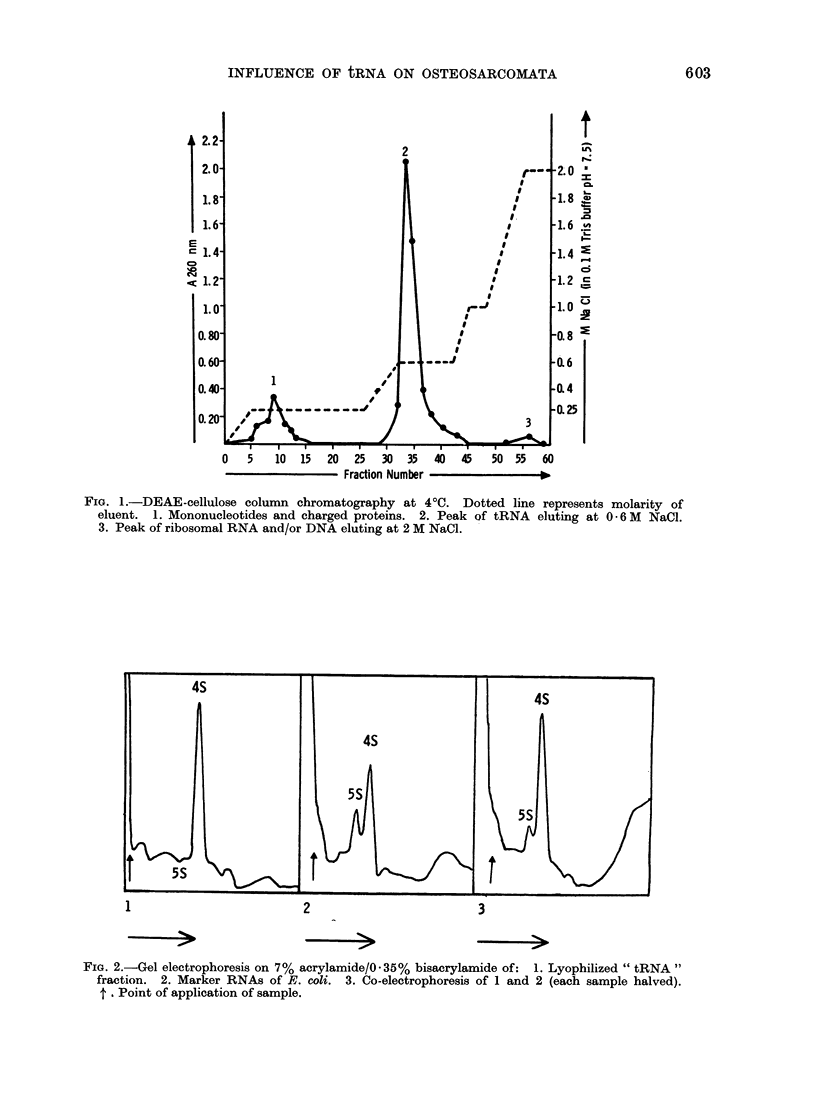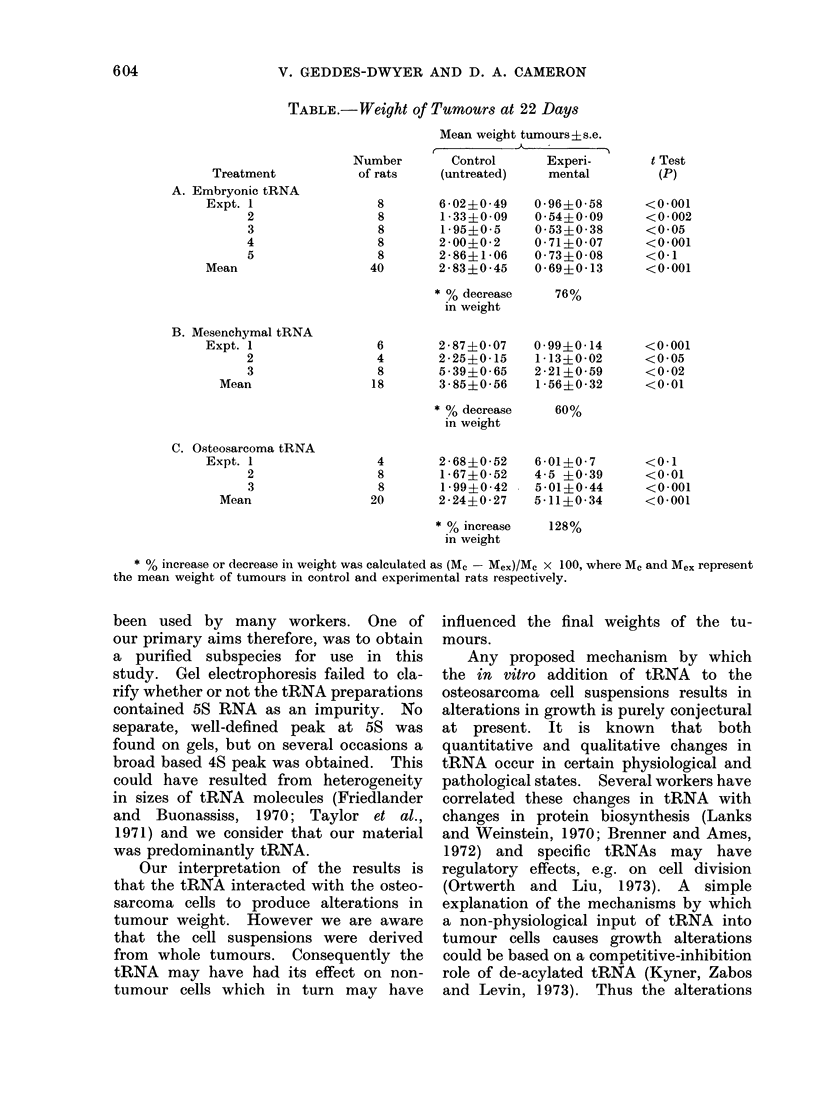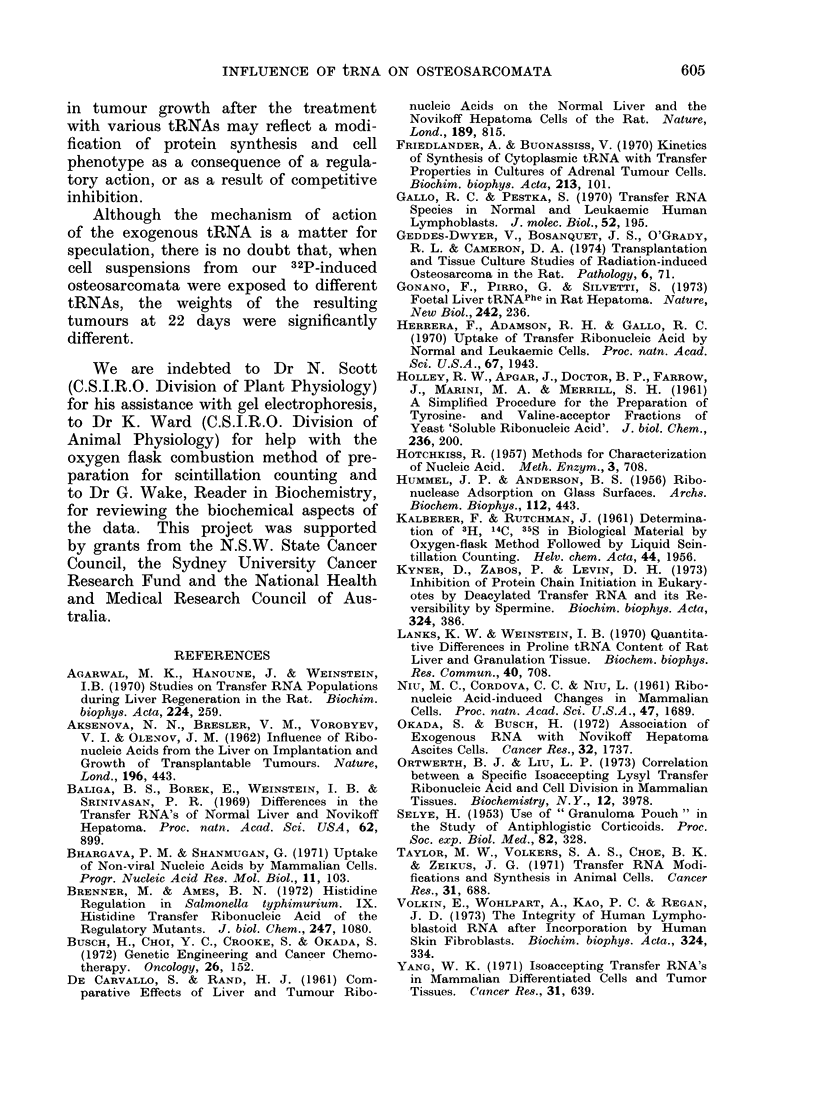# Influence of exogenous tRNA on growth of transplantable 32P-induced osteosarcomata.

**DOI:** 10.1038/bjc.1976.97

**Published:** 1976-06

**Authors:** V. Geddes-Dwyer, D. A. Cameron

## Abstract

The weights of osteosarcomata that arose 22 days after the s.c. injection of cell suspensions (of whold tumours) that had been exposed in vitro to tRNA were significantly different from the weights of those arising from untreated cells. The tRNA was isolated by phenol extraction and DEAE-cellulose chromatography from eviscerated full-term rat embryos, from rat mesenchymal (granulation) tissue and from rat anaplastic osteosarcomata. Tumours developing from osteosarcoma cells treated with either embryonic or normal mesenchymal tRNA were reduced in weight by 76% and 60% respectively. These effects could not be explained as a toxic consequence, because tumour weight was increased by 128% after exposure of cells to osteosarcoma tRNA. In this osteosarcoma model it appears that tumour weights can be influenced in different ways by tRNA from different sources.


*Br. J. Cancer* (1976) **33**, 600

### INFLUENCE OF EXOGENOUS tRNA ON GROWTH OF TRANSPLANTABLE $^{32}$P-INDUCED OSTEOSARCOMATA

V. GEDDES-DWYER AND D. A. CAMERON

*From the Department of Pathology, University of Sydney, N.S.W., 2006, Australia*

Received 8 December 1975 Accepted 10 February 1976

**Summary.—The weights of osteosarcomata that arose 22 days after the s.c. injection of cell suspensions (of whole tumours) that had been exposed *in vitro* to tRNA were significantly different from the weights of those arising from untreated cells. The tRNA was isolated by phenol extraction and DEAE-cellulose chromatography from eviscerated full-term rat embryos, from rat mesenchymal (granulation) tissue and from rat anaplastic osteosarcomata. Tumours developing from osteosarcoma cells treated with either embryonic or normal mesenchymal tRNA were reduced in weight by 76% and 60% respectively. These effects could not be explained as a toxic consequence, because tumour weight was increased by 128% after exposure of cells to osteosarcoma tRNA. In this osteosarcoma model it appears that tumour weights can be influenced in different ways by tRNA from different sources.**

IT IS KNOWN that tumour and other cells can effectively take up transfer ribonucleic acids (tRNA) *in vitro*, and that an appreciable percentage of this retains its polymeric form for some hours and remains functional, i.e., able to accept amino acids (Herrera, Adamson and Gallo, 1970; Busch *et al.*, 1972; Volkin *et al.*, 1973).

In view of reports of differences either in the amount of one isoaccepting tRNA species or in the number of isoaccepting species in various tissues during embryogenesis (Taylor *et al.*, 1971) and regeneration (Agarwal, Hanoune and Weinstein, 1970) or oncogenesis (Baliga *et al.*, 1969), it is conceivable that changes in tRNA may be crucial for maintaining a cell in a particular state of differentiation.

Several workers have reported that the addition to tumour cells of exogenous RNA, extracted from normal tissues, could inhibit their growth (De Carvallo and Rand, 1961; Niu, Cordova and Niu, 1961; Aksenova *et al.*, 1962). Recent investigations of uptake of RNA have shown that recipient cells preferentially take up tRNA (Volkin *et al.*, 1973) and that the degree of degradation of 4S RNA is less than that of the other types of RNA studied (Okada and Busch, 1972). It therefore seems possible that tRNA is the sole causative agent of such documented effects of exogenous RNA. The present work was undertaken to investigate the effect of the addition of exogenous tRNA to cell suspensions of osteosarcomata on their subsequent growth.

Granulation tissue was selected as a source of normal mesenchymal tRNA, because it provided this nucleic acid in quantity without the technical problems associated with the use of muscle or bone. Embryonic tRNA was used because of the revived interest in the concept of depression, in neoplasia, of genes that normally function only in the embryonic tissue of origin and because embryonic-like tRNA species have been found in malignant cells (Yang, 1971; Gonano,

Pirro and Silvetti, 1973). Transfer RNA derived from anaplastic osteosarcomata was used to determine whether or not a " double dose " of the tRNA species present in the tumour cells would enhance growth.

### MATERIALS AND METHODS

*Sources of tRNA.*—Transfer RNA was isolated and purified from rat embryonic tissue, from normal rat mesenchymal tissue and from a rat non-bone-forming osteosarcoma. The full-term embryos were eviscerated to ensure the greatest yield of mesenchymal tRNA. The mesenchymal tissue was obtained from granuloma pouches, produced by a 0·1 ml injection of 1% croton oil suspended in corn oil (Selye, 1953).

*Tumours.*—Osteosarcomata were of the 20th to 41st transplant generations of a tumour induced in an inbred DA rat by $^{32}P$ as previously described (Geddes-Dwyer *et al.*, 1974). They were non-calcifying, very cellular with a minimum of matrix, and transplanted at 3–4 week intervals depending on experimental requirements.

*Preparation of tRNA.*—The following precautions were taken to reduce the possibility of degradation of tRNA during preparation. (a) Because ribonuclease has a high affinity for glass (Hummel and Anderson, 1956) all glassware was cleaned, either with 30% hydrogen peroxide, or when size permitted, by steam sterilization. (b) Polyethylene gloves were worn during all procedures. (c) After rapid aseptic removal, tissue was dropped immediately into liquid air. (d) Analytical reagents only were used and unless otherwise mentioned, all isolation and purification procedures were performed at a constant pH of 7·5 at 4°C.

(i) *Extraction of tRNA*

Twenty g of frozen tissue was homogenized in a Waring Blender for 10 min with 30 ml of 1·0 M NaCl, 0·005 M EDTA in 0·1 M Tris-chloride buffer at pH 7·5 and 30 ml phenol saturated with this solution. The homogenate was centrifuged for 20 min at 5000 *g*. The aqueous phase was removed with a sterile pipette, mixed with an equal amount of buffer-saturated phenol, and re-centrifuged as above. The aqueous layer was carefully pipetted off. Three volumes of 95% ethanol containing 2% potassium acetate were added to it and any DNA present was spooled out as a rapidly forming precipitate. After a minimum of 2 h at −20°C the nucleic acid flocculated in the remaining aqueous layer/ethanol solution.

(ii) *DEAE-cellulose chromatography*

The tRNA was further purified by ion-exchange chromatography (Holley *et al.*, 1961). The sample to be chromatographed was dissolved in 0·1 M Tris-buffer and applied to a squat DEAE-cellulose column (8 cm) (Whatman DE-23) with a diameter of 2 cm. All contaminating polysaccharides and neutral proteins were washed off with 1·5 l of 0·1 M Tris-buffer at pH 7·5. Stepwise elution was then performed using 0·25 M, 0·6 M, 1 M and 2 M NaCl in 0·2 M Tris-buffer (pH 7·5). UV analysis was carried out on the eluted samples and fractions containing the tRNA, which elutes at 0·6 M NaCl (Fig. 1), were precipitated with 95% ethanol/2% potassium acetate and left overnight at −20°C to flocculate maximally. The precipitate was spun down and washed first in 80% ethanol and then twice in 95% and lyophilized. The concentration of tRNA was estimated on the basis of one optical density unit at 260 nm being equivalent to 45 μg (Hotchkiss, 1975). The yield of tRNA was approximately 0·5 mg/g of tissue extracted.

*Gel electrophoresis of lyophilized fraction.*—This was carried out on both 12% and 7% acrylamide/0·35% bisacrylamide at 50 V and 3 mA per gel for 6 h in 0·12 M Tris/0·05 M NaAc/0·003 M EDTA at pH 7·8. *E. coli* RNAs were used as markers in order to establish the sedimentation velocity of this fraction.

*Assay of tRNA amino acid-accepting activity.*—Both the preparation of amino-acyl synthetase and the assay were performed according to the methods described by Gallo and Pestka (1970) with the following minor modifications: (i) tRNA was removed from all amino-acyl tRNA synthetase preparations by precipitation with 5% streptomycin sulphate; (ii) endogenous amino acids were removed by Sephadex G25 chromatography; (iii) protein hydrolysate −$^{14}C$ (Amersham) with a specific activity of 57 μCi/milli-atom carbon was used as a source of uniformly labelled amino acids in the assay; (iv) the precipitates, after being washed with 5% trichloracetic acid and dried with an infra-red lamp on the HA Millipore filters were counted in a scintillant cocktail (toluene : PPO : POPOP : 100 ml : 400 mg : 2 mg). Counting on filters was checked by the oxygen flask combustion method (Kalberer and Rutchmann, 1961) and comparable results were obtained.

*Preparation and treatment of cells.*—Cells that were to be exposed to the tRNA were separated enzymatically with pronase (2 mg/ml, Calbiochem) and DNAase (125 $\mu$g/ml, Sigma—DN25). Between $10^7$ and $10^8$ cells/g of tumour tissue were obtained.

The lyophilized tRNA was dissolved in Eagle's medium and added at a concentration of 500 $\mu$g/ml (Herrera *et al.*, 1970) to an aliquot of tumour cells ($2 \times 10^6$ cells/ml) suspended in Eagle's medium without serum and buffered with Hepes. This mixture was left on an orbital shaker at 4°C overnight. Two controls, one containing Eagle's medium and cells only and the other containing cells plus degradation products from RNAase digestion of tRNA, were similarly treated. In the latter control, tRNA (500 $\mu$g/ml) had been incubated with RNAase (Calbiochem–bovine pancreas, 100 $\mu$g/ml) at 37°C for 10 min and the resultant breakdown products were then added to the aliquot of tumour cells. Cell numbers or viability did not drop in either the control or experimental aliquots after this 15 h incubation.

The treated and the control cells were spun down, washed and re-counted. Each aliquot was subdivided into 0·5 ml quantities containing $2 \times 10^6$ cells for deep s.c. injection into the flank of syngeneic rats of the same sex and weight for the control and experimental series. Between 4 and 8 rats were used in each experimental series. After 22 days all animals were killed and the tumours removed and weighed. Eleven separate experiments, utilizing freshly prepared tRNA in each instance, were carried out.

### RESULTS

### *Purity of tRNA preparation*

Any mononucleotides or protein contaminating the tRNA preparation applied to the DEAE-cellulose column were eluted with 0·25 M NaCl in the Tris buffer (indicated by the first peak in Fig. 1). The 260/280 ratio (u.v. analysis) of the tRNA fraction eluted with 0·6 M NaCl was always $\geq 2$. No protein was detected by the Lowry method of estimation. Although there may have been some protein present, it appeared to have had no effect, as was shown by the unaltered behaviour of cells exposed to the RNAase-treated preparation.

Co-electrophoresis of the lyophilized fraction with *E. coli* RNA markers on the gels showed that the peak obtained coincided with the 4S RNA of *E. coli* (Fig. 2). Electrophoresis on 12% acrylamide gels showed that there was no contamination of the fraction with either DNA or high molecular weight RNA. Calculation showed that the tRNA was functional with approximately 80% of the preparation aminoacylated.

### *Effect of exogenous tRNA on tumour weights*

Tumours derived from osteosarcoma cell suspensions exposed to either the embryonic or mesenchymal tissue tRNA weighed less at the end of the 22-day experimental period (76% and 60% respectively) than those derived from control cells. Tumours developing from osteosarcoma cell suspensions exposed to tRNA derived from an osteosarcoma grew much larger (128%) than those from the cells without such treatment. Student's *t* test of all the data for each treatment showed that the differences between control and experimental were significant (Table).

Tumours from osteosarcoma cells exposed to tRNA degraded with pancreatic RNAase were not significantly different in weight from the weights of tumours from untreated cells.

### DISCUSSION

Interpretation of the many biological and biochemical effects ascribed to exogenous RNA (Bhargava and Shanmugan, 1971) has been made difficult because total RNA or a mixture of RNAs have

FIG. 1.—DEAE-cellulose column chromatography at 4°C. Dotted line represents molarity of eluent. 1. Mononucleotides and charged proteins. 2. Peak of tRNA eluting at 0·6 M NaCl. 3. Peak of ribosomal RNA and/or DNA eluting at 2 M NaCl.

FIG. 2.—Gel electrophoresis on 7% acrylamide/0·35% bisacrylamide of: 1. Lyophilized " tRNA " fraction. 2. Marker RNAs of *E. coli*. 3. Co-electrophoresis of 1 and 2 (each sample halved). ↑. Point of application of sample.

TABLE.—*Weight of Tumours at 22 Days*

| Treatment | Number of rats | Mean weight tumours ± s.e. Control (untreated) | Experimental | t Test (P) |
|---|---|---|---|---|
| A. Embryonic tRNA | | | | |
| Expt. 1 | 8 | 6·02±0·49 | 0·96±0·58 | <0·001 |
| 2 | 8 | 1·33±0·09 | 0·54±0·09 | <0·002 |
| 3 | 8 | 1·95±0·5 | 0·53±0·38 | <0·05 |
| 4 | 8 | 2·00±0·2 | 0·71±0·07 | <0·001 |
| 5 | 8 | 2·86±1·06 | 0·73±0·08 | <0·1 |
| Mean | 40 | 2·83±0·45 | 0·69±0·13 | <0·001 |
| | | * % decrease in weight | 76% | |
| B. Mesenchymal tRNA | | | | |
| Expt. 1 | 6 | 2·87±0·07 | 0·99±0·14 | <0·001 |
| 2 | 4 | 2·25±0·15 | 1·13±0·02 | <0·05 |
| 3 | 8 | 5·39±0·65 | 2·21±0·59 | <0·02 |
| Mean | 18 | 3·85±0·56 | 1·56±0·32 | <0·01 |
| | | * % decrease in weight | 60% | |
| C. Osteosarcoma tRNA | | | | |
| Expt. 1 | 4 | 2·68±0·52 | 6·01±0·7 | <0·1 |
| 2 | 8 | 1·67±0·52 | 4·5 ±0·39 | <0·01 |
| 3 | 8 | 1·99±0·42 | 5·01±0·44 | <0·001 |
| Mean | 20 | 2·24±0·27 | 5·11±0·34 | <0·001 |
| | | * % increase in weight | 128% | |

* % increase or decrease in weight was calculated as $(M_c - M_{ex})/M_c \times 100$, where $M_c$ and $M_{ex}$ represent the mean weight of tumours in control and experimental rats respectively.

been used by many workers. One of our primary aims therefore, was to obtain a purified subspecies for use in this study. Gel electrophoresis failed to clarify whether or not the tRNA preparations contained 5S RNA as an impurity. No separate, well-defined peak at 5S was found on gels, but on several occasions a broad based 4S peak was obtained. This could have resulted from heterogeneity in sizes of tRNA molecules (Friedlander and Buonassiss, 1970; Taylor *et al.*, 1971) and we consider that our material was predominantly tRNA.

Our interpretation of the results is that the tRNA interacted with the osteosarcoma cells to produce alterations in tumour weight. However we are aware that the cell suspensions were derived from whole tumours. Consequently the tRNA may have had its effect on non-tumour cells which in turn may have influenced the final weights of the tumours.

Any proposed mechanism by which the *in vitro* addition of tRNA to the osteosarcoma cell suspensions results in alterations in growth is purely conjectural at present. It is known that both quantitative and qualitative changes in tRNA occur in certain physiological and pathological states. Several workers have correlated these changes in tRNA with changes in protein biosynthesis (Lanks and Weinstein, 1970; Brenner and Ames, 1972) and specific tRNAs may have regulatory effects, e.g. on cell division (Ortwerth and Liu, 1973). A simple explanation of the mechanisms by which a non-physiological input of tRNA into tumour cells causes growth alterations could be based on a competitive-inhibition role of de-acylated tRNA (Kyner, Zabos and Levin, 1973). Thus the alterations in tumour growth after the treatment with various tRNAs may reflect a modification of protein synthesis and cell phenotype as a consequence of a regulatory action, or as a result of competitive inhibition.

Although the mechanism of action of the exogenous tRNA is a matter for speculation, there is no doubt that, when cell suspensions from our $^{32}P$-induced osteosarcomata were exposed to different tRNAs, the weights of the resulting tumours at 22 days were significantly different.

We are indebted to Dr N. Scott (C.S.I.R.O. Division of Plant Physiology) for his assistance with gel electrophoresis, to Dr K. Ward (C.S.I.R.O. Division of Animal Physiology) for help with the oxygen flask combustion method of preparation for scintillation counting and to Dr G. Wake, Reader in Biochemistry, for reviewing the biochemical aspects of the data. This project was supported by grants from the N.S.W. State Cancer Council, the Sydney University Cancer Research Fund and the National Health and Medical Research Council of Australia.

### REFERENCES

AGARWAL, M. K., HANOUNE, J. & WEINSTEIN, I.B. (1970) Studies on Transfer RNA Populations during Liver Regeneration in the Rat. *Biochim. biophys. Acta*, **224**, 259.

AKSENOVA, N. N., BRESLER, V. M., VOROBYEV, V. I. & OLENOV, J. M. (1962) Influence of Ribonucleic Acids from the Liver on Implantation and Growth of Transplantable Tumours. *Nature, Lond.*, **196**, 443.

BALIGA, B. S., BOREK, E., WEINSTEIN, I. B. & SRINIVASAN, P. R. (1969) Differences in the Transfer RNA's of Normal Liver and Novikoff Hepatoma. *Proc. natn. Acad. Sci. USA*, **62**, 899.

BHARGAVA, P. M. & SHANMUGAN, G. (1971) Uptake of Non-viral Nucleic Acids by Mammalian Cells. *Progr. Nucleic Acid Res. Mol. Biol.*, **11**, 103.

BRENNER, M. & AMES, B. N. (1972) Histidine Regulation in *Salmonella typhimurium*. IX. Histidine Transfer Ribonucleic Acid of the Regulatory Mutants. *J. biol. Chem.*, **247**, 1080.

BUSCH, H., CHOI, Y. C., CROOKE, S. & OKADA, S. (1972) Genetic Engineering and Cancer Chemotherapy. *Oncology*, **26**, 152.

DE CARVALLO, S. & RAND, H. J. (1961) Comparative Effects of Liver and Tumour Ribonucleic Acids on the Normal Liver and the Novikoff Hepatoma Cells of the Rat. *Nature, Lond.*, **189**, 815.

FRIEDLANDER, A. & BUONASSISS, V. (1970) Kinetics of Synthesis of Cytoplasmic tRNA with Transfer Properties in Cultures of Adrenal Tumour Cells. *Biochim. biophys. Acta*, **213**, 101.

GALLO, R. C. & PESTKA, S. (1970) Transfer RNA Species in Normal and Leukaemic Human Lymphoblasts. *J. molec. Biol.*, **52**, 195.

GEDDES-DWYER, V., BOSANQUET, J. S., O'GRADY, R. L. & CAMERON, D. A. (1974) Transplantation and Tissue Culture Studies of Radiation-induced Osteosarcoma in the Rat. *Pathology*, **6**, 71.

GONANO, F., PIRRO, G. & SILVETTI, S. (1973) Foetal Liver $tRNA^{Phe}$ in Rat Hepatoma. *Nature, New Biol.*, **242**, 236.

HERRERA, F., ADAMSON, R. H. & GALLO, R. C. (1970) Uptake of Transfer Ribonucleic Acid by Normal and Leukaemic Cells. *Proc. natn. Acad. Sci. U.S.A.*, **67**, 1943.

HOLLEY, R. W., APGAR, J., DOCTOR, B. P., FARROW, J., MARINI, M. A. & MERRILL, S. H. (1961) A Simplified Procedure for the Preparation of Tyrosine- and Valine-acceptor Fractions of Yeast 'Soluble Ribonucleic Acid'. *J. biol. Chem.*, **236**, 200.

HOTCHKISS, R. (1957) Methods for Characterization of Nucleic Acid. *Meth. Enzym.*, **3**, 708.

HUMMEL, J. P. & ANDERSON, B. S. (1956) Ribonuclease Adsorption on Glass Surfaces. *Archs. Biochem. Biophys.*, **112**, 443.

KALBERER, F. & RUTCHMAN, J. (1961) Determination of $^{3}H$, $^{14}C$, $^{35}S$ in Biological Material by Oxygen-flask Method Followed by Liquid Scintillation Counting. *Helv. chem. Acta*, **44**, 1956.

KYNER, D., ZABOS, P. & LEVIN, D. H. (1973) Inhibition of Protein Chain Initiation in Eukaryotes by Deacylated Transfer RNA and its Reversibility by Spermine. *Biochim. biophys. Acta*, **324**, 386.

LANKS, K. W. & WEINSTEIN, I. B. (1970) Quantitative Differences in Proline tRNA Content of Rat Liver and Granulation Tissue. *Biochem. biophys. Res. Commun.*, **40**, 708.

NIU, M. C., CORDOVA, C. C. & NIU, L. (1961) Ribonucleic Acid-induced Changes in Mammalian Cells. *Proc. natn. Acad. Sci. U.S.A.*, **47**, 1689.

OKADA, S. & BUSCH, H. (1972) Association of Exogenous RNA with Novikoff Hepatoma Ascites Cells. *Cancer Res.*, **32**, 1737.

ORTWERTH, B. J. & LIU, L. P. (1973) Correlation between a Specific Isoaccepting Lysyl Transfer Ribonucleic Acid and Cell Division in Mammalian Tissues. *Biochemistry, N.Y.*, **12**, 3978.

SELYE, H. (1953) Use of " Granuloma Pouch " in the Study of Antiphlogistic Corticoids. *Proc. Soc. exp. Biol. Med.*, **82**, 328.

TAYLOR, M. W., VOLKERS, S. A. S., CHOE, B. K. & ZEIKUS, J. G. (1971) Transfer RNA Modifications and Synthesis in Animal Cells. *Cancer Res.*, **31**, 688.

VOLKIN, E., WOHLPART, A., KAO, P. C. & REGAN, J. D. (1973) The Integrity of Human Lymphoblastoid RNA after Incorporation by Human Skin Fibroblasts. *Biochim. biophys. Acta.*, **324**, 334.

YANG, W. K. (1971) Isoaccepting Transfer RNA's in Mammalian Differentiated Cells and Tumor Tissues. *Cancer Res.*, **31**, 639.